# The Perfect Cytokine Storm: Utilization of Lung Ultrasound During Urgent Surgery in an Infant With Multisystem Inflammatory Syndrome in Children and Hemophagocytic Lymphohistiocytosis

**DOI:** 10.7759/cureus.15640

**Published:** 2021-06-14

**Authors:** Michael A Waterhouse, Anrew Villion, Toni Manougian, Irim Salik

**Affiliations:** 1 Anesthesiology, Westchester Medical Center, Valhalla, USA; 2 Critical Care Medicine, Westchester Medical Center, Valhalla, USA; 3 Pediatric Anesthesiology, Westchester Medical Center, Valhalla, USA

**Keywords:** covid-19, hemophagocytic lymphohistiocytosis (hlh), lung ultrasound (lus), mis-c, pediatric critical care medicine, intraoperative ultrasound

## Abstract

Reports of children with a temporal association to severe acute respiratory syndrome coronavirus-2 hospitalized with cardiogenic shock or Kawasaki-like disease began emerging in April 2020. In May 2020, the Centers for Disease Control and Prevention published the criteria for what came to be known as multisystem inflammatory syndrome in children, a postinfectious inflammatory immune response to coronavirus disease 2019 exposure. Hemophagocytic lymphohistiocytosis is a heterogeneous disease state associated with systemic hyperinflammation secondary to immune dysregulation. We describe the utility of perioperative lung ultrasound in an infant with both disease states.

## Introduction

Multisystem inflammatory syndrome in children (MIS-C) is a postinfectious inflammatory immune response to coronavirus disease 2019 (COVID-19) exposure. In May 2020, the Centers for Disease Control and Prevention published the criteria for the definition of MIS-C due to COVID-19 exposure [1]. While clinical manifestations, treatment options, and long-term sequelae have been increasingly identified, the overlap of MIS-C and other “pro-inflammatory” syndromes has not been well elucidated. It is not yet evident whether hemophagocytic lymphohistiocytosis (HLH) can occur simultaneously and independently of MIS-C, or as a direct consequence of infection with severe acute respiratory syndrome coronavirus-2 (SARS-CoV-2). HLH is characterized as either a familial autosomal recessive disorder, or acquired due to immunodeficiency, infection, or underlying malignancy [2]. A cytokine storm results from tissue infiltration of lymphocytes and macrophages. There is significant overlap between the features reported in MIS-C and HLH diagnostic criteria, with a prominent etiology of monocytes and macrophages in the pathogenesis of viral infection for both syndromes. We discuss the utilization of lung ultrasound (LUS) for anesthetic management in a complex infant with severe acute respiratory distress syndrome (ARDS) who presented with MIS-C and HLH concurrently.

## Case presentation

**Figure 1:**
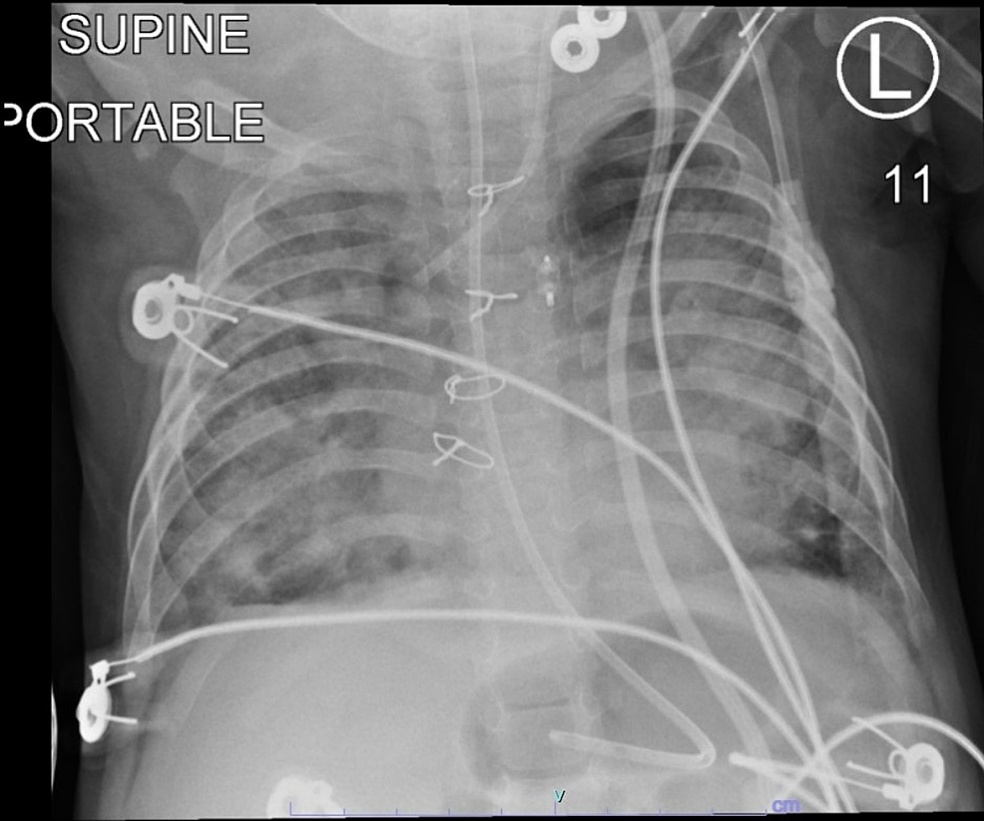
Chest X-ray demonstrating extensive consolidation superimposed on chronic interstitial markings secondary to bronchopulmonary dysplasia.

The patient is an 11-month-old female, ex-35-week gestational age, with Down syndrome, a history of a ventricular septal defect corrected at three months of age, and pulmonary hypertension on sildenafil and oxygen via nasal cannula. She presented at six months of age with a diffuse rash, vomiting, severe thrombocytopenia, fever, and tachypnea. She was positive for COVID-19 via polymerase chain reaction and completed a course of remdesivir, dexamethasone, convalescent plasma, and intravenous immunoglobulin, with a presumptive diagnosis of MIS-C. She subsequently developed worsening pancytopenia, hyperferritinemia, and hypertriglyceridemia; a bone marrow biopsy revealed hemophagocytosis, and she was diagnosed with HLH. She was started on anakinra, etoposide, and gamifant due to refractory HLH. She continued to require hospitalization over the next few months with waxing and waning clinical symptoms. Prior to her 11-month birthday, the infant developed worsening respiratory distress requiring bi-level positive airway pressure (BiPAP) support. A chest X-ray showed evidence of aspiration pneumonia (Figure 1), and she was scheduled for urgent gastrostomy tube placement.

On the day of the surgery, the infant weighed 6.3 kg and was on BiPAP 12/8/40% FiO_2_ with an SpO_2_ of 91%. The infant required frequent platelet transfusions with a recent nadir of 29,000/mm^3^ platelets. She tested negative for COVID-19 as per the hospital protocol. She was currently receiving infusions of intravenous piperacillin/tazobactam for aspiration pneumonia and oral vancomycin for *Clostridium difficile* infection. The patient was maintained on sildenafil, spironolactone, and inhaled nitric oxide for worsening pulmonary hypertension, likely due to pulmonary aspiration and the pulmonary manifestations of MIS-C. Anesthesia was induced with propofol 1 mg/kg, dexmedetomidine 0.5 mcg/kg, and rocuronium 1.2 mg/kg. Intubation was performed with video laryngoscopy, and a 4.0 cuffed endotracheal tube was placed without difficulty.

**Figure 2:**
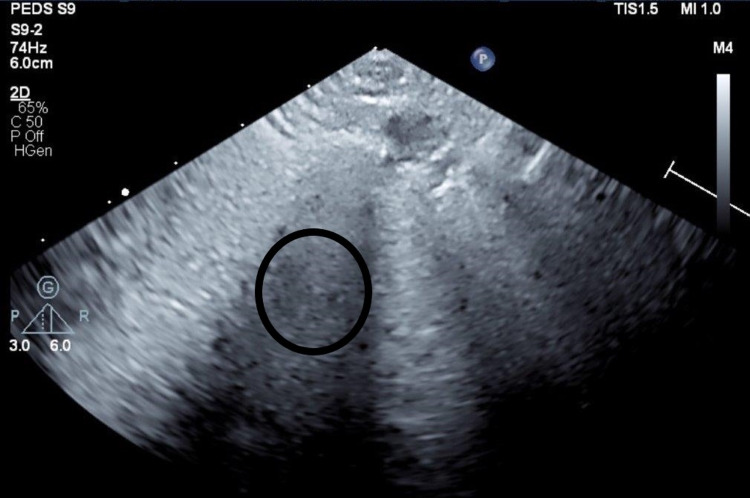
Lung ultrasound revealing obliteration of A-lines, confluence of B-lines (bright “comets”), and pulmonary hepatization (circled).

The patient was difficult to mechanically ventilate, requiring escalating peak inspiratory pressure and positive end-expiratory pressure to maintain an SpO_2_ of 85%. The patient required 10 mL/kg each of packed red blood cells and platelets. LUS was performed once the gastrostomy tube was in place to evaluate the patient’s deteriorating pulmonary status (Figure 2).

LUS revealed dense lung fields with hyperechoic signals, coalesced B-lines, and pulmonary hepatization. This was interpreted as severe interstitial edema with the hyperdense areas signifying consolidation and severe ARDS. The patient was switched to airway pressure release ventilation (APRV), which has shown efficacy in pediatric ARDS patients (PHigh 25 cm H_2_O, PLow 0 cm H_2_O, THigh 5 s, TLow 0.5 s) [3]. Oxygenation and hemodynamic parameters improved throughout the case. Upon completion of the case, the patient was left intubated secondary to these findings, and the intensive care unit (ICU) was counseled to continue APRV postoperatively. Over the course of the next few months, the patient continued to deteriorate from multiorganism sepsis, worsening of her inflammatory state, and cardiopulmonary compromise. She eventually expired three months following surgical intervention.

## Discussion

In patients with HLH, activated macrophages engulf erythrocytes, platelets, and leukocytes along with their precursor cells in lymph nodes, the spleen, peripheral blood, and the bone marrow. Clinically, it presents as a multisystemic disease associated with persistent fevers, progressive cytopenia, and immune-mediated end-organ dysfunction. Acquired HLH can complicate the underlying disease, and it is frequently associated with viral infections, including Epstein-Barr, cytomegalovirus, adenovirus, and influenza [4]. It stands to reason that SARS-CoV-2 may also be considered to be a potential etiologic trigger for HLH. It is common for patients with HLH to not fulfill at least five of the following diagnostic criteria: fever, splenomegaly, peripheral blood cytopenia, hypertriglyceridemia, hemophagocytosis, reduced natural killer (NK) cell activity, hyperferritinemia, and elevated soluble CD25 levels. In pediatric patients, HLH is usually caused by germline mutations impairing granulocyte-mediated cytotoxicity. Initial signs and symptoms can mimic common infections, including hypogammaglobulinemia, diarrhea, bleeding, and sensorineural hearing loss [5].

In adult patients, the frequency of HLH in patients with severe COVID-19 is less than 5%, although this may be an underestimation based upon the lack of histopathologic criteria reporting. In patients diagnosed with COVID-19 who present with high fever, hepatosplenomegaly, progressive pancytopenia, or the HLH triad (hyperferritinemia, hypertriglyceridemia, or hypofibrinogenemia), HLH should be ruled out. These patients should be evaluated with immunologic markers and histopathologic studies. Gómez-Rial et al. [6] have indicated a pathogenic role of exaggerated monocyte and macrophage activation in COVID-19 that is also seen in other hyperinflammatory syndromes, such as HLH. Furthermore, Gustine et al. [7] reported that the hyperinflammatory syndrome associated with COVID-19 may have pathogenic overlap with viral-induced HLH secondary to macrophage activation and a cytokine storm with impairment of NK cells and CD8+ T cells.

Fever, lymphopenia, thrombocytopenia, and anemia are among the features shared by severe COVID-19 inflammatory storm and HLH. Lymphopenia in COVID-19 patients has been a prognostic indicator of ARDS development, the need for ICU admission, and reduced survival [8]. In contrast to HLH, pancytopenia is seen in only 3-4% of patients with COVID-19. Furthermore, fibrinogen levels are usually elevated due to increased interleukin-6 activity causing fibrinogen synthesis. Patients who become critically ill may develop low fibrinogen levels prior to progression to disseminated intravascular coagulation or HLH [9]. Although there is no definitive threshold to differentiate HLH from COVID-19, ferritin levels higher than 10,000 ng/mL are more likely to indicate HLH. In a sample of 60 patients with severe COVID-19 in whom HLH was confirmed, only 13% fulfilled at least five of the HLH diagnostic criteria [10].

Several authors have suggested that COVID-19 be included in the umbrella of “hyperferritinemic syndromes,” including systemic juvenile idiopathic arthritis, adult-onset Still’s disease, antiphospholipid syndrome, macrophage activation syndrome (MAS), and HLH, all associated with a high mortality rate [7]. The frequency of MAS is higher in pediatric patients with severe MIS-C than in adults, with 25% of 118 children identified in a European study [11]. MIS-C uniquely displays features of type II hypersensitivity reactions (oligoclonal immunoglobulin A plasma cells in arteries of children with Kawasaki-like disease) and type IV hypersensitivity reactions (HLH or MAS) [9].

There are numerous anesthetic considerations in patients with HLH and MIS-C concurrently. Coagulopathic patients may present with an increased partial thromboplastin time, while hyperbilirubinemia can often lead to jaundice. Failure to thrive, malaise, and lymphadenopathy can complicate a diagnosis of HLH. Neurologic abnormalities, including seizures, may occur in patients. In MIS-C patients with HLH, anesthesiologists should be cognizant of the increased risk of anemia, hemorrhage, infection, multiorgan dysfunction, acute lung injury, or ARDS. Preoperative laboratory evaluation should include a complete blood count and liver function tests. Strict asepsis is indicated in these patients, and prophylactic antibiotics should take immunologic status into consideration. Coagulopathy, anemia, and thrombocytopenia should be corrected prior to anesthetic induction. The risks and benefits of a regional anesthetic should be weighed in a patient with infection or coagulopathy. Administration of hepatotoxic medications should be avoided in patients with hepatic dysfunction.

Lung protective strategies should be employed in pediatric patients with ARDS. Although APRV is widely utilized in adult critical care settings, its use in pediatric patients is largely limited to children with ARDS. APRV utilizes a long period of continuous positive pressure (PHigh) for a period of several seconds (THigh) with a low level of CPAP (PLow) for a shorter period (TLow) [3]. APRV enables CO_2_ elimination and allows for lung recruitment without additional expiratory pressure. Mean airway pressure is increased without an elevation in plateau pressure. In a spontaneously breathing patient, right atrial (RA) pressure is lowered and inferior vena cava (IVC) blood flow is increased via decreased pleural pressure. In ARDS patients, an increased pressure gradient between the RA and IVC leads to increased venous return and improved cardiac output [3].

LUS is a useful tool for real-time visualization of the air-fluid interface in patients with severe ARDS. Pulmonary hepatization refers to the acoustic impedance of ultrasound waves by alveolar infiltration of inflammatory exudates, resembling the liver. In an ARDS patient, LUS demonstrates heterogeneous alveolar-interstitial syndrome, multiple bilateral lung regions with two or more B-lines, and bilateral consolidations [12]. A study by Lichtenstein et al. [13] found that LUS demonstrated increased diagnostic accuracy for ARDS in comparison to chest radiography, with thoracic computed tomography as the gold standard.

## Conclusions

It is important to maintain high clinical suspicion for HLH in patients with MIS-C who present with clinical and laboratory findings including coagulopathy, hepatosplenomegaly, cytokinemia, lymphopenia, thrombocytopenia, and macrophage activation. In a critically ill patient with MIS-C and HLH, anesthetic management for surgical intervention is an endeavor fraught with pitfalls and challenges. In the case of this complex infant who presented with MIS-C and HLH in tandem, LUS was useful in the diagnosis and management of ARDS.
